# Repurposing dextromethorphan and metformin for treating nicotine-induced cancer by directly targeting CHRNA7 to inhibit JAK2/STAT3/SOX2 signaling

**DOI:** 10.1038/s41388-021-01682-z

**Published:** 2021-02-18

**Authors:** Lu Wang, Liang Du, Xiao Xiong, Yusheng Lin, Jianlin Zhu, Zhimeng Yao, Shuhong Wang, Yi Guo, Yuping Chen, Kyla Geary, Yunlong Pan, Fuyou Zhou, Shegan Gao, Dianzheng Zhang, Sai-Ching Jim Yeung, Hao Zhang

**Affiliations:** 1 Department of General Surgery, The First Affiliated Hospital of Jinan University, and Institute of Precision Cancer Medicine and Pathology, School of Medicine, Jinan University, Guangzhou, Guangdong China; 2 Department of Biomedical Sciences of Cells & Systems, Section Molecular Cell Biology and Radiation Oncology, University Medical Center Groningen, University of Groningen, Groningen, The Netherlands; 3 Department of Hematology, University Medical Center Groningen, University of Groningen, Groningen, The Netherlands; 4 Endoscopy Center, Affiliated Cancer Hospital of Shantou University Medical College, Shantou, Guangdong China; 5 Department of Thoracic Surgery, Affiliated Cancer Hospital of Shantou University Medical College, Shantou, Guangdong China; 6 Department of Bio-Medical Sciences, Philadelphia College of Osteopathic Medicine, 4170 City Avenue, Philadelphia, PA 19131 USA; 7 The Fourth Affiliated Hospital of Henan University of Science and Technology, Anyang, 455001 Henan China; 8 Department of Thoracic Surgery, Anyang Tumor Hospital, Anyang, 455001 Henan China; 9 College of Clinical Medicine, The First Affiliated Hospital of Henan University of Science and Technology, Henan Key Laboratory of Cancer Epigenetics, Luoyang, 471003 China; 10 Department of Emergency Medicine, University of Texas MD Anderson Cancer Center, Houston, TX USA; 11 Department of Endocrine Neoplasia and Hormonal Disorders, University of Texas MD Anderson Cancer Center, Houston, TX USA

**Keywords:** Cancer prevention, Drug development, Targeted therapies, Oesophageal cancer, Oncogenes

## Abstract

Smoking is one of the most impactful lifestyle-related risk factors in many cancer types including esophageal squamous cell carcinoma (ESCC). As the major component of tobacco and e-cigarettes, nicotine is not only responsible for addiction to smoking but also a carcinogen. Here we report that nicotine enhances ESCC cancer malignancy and tumor-initiating capacity by interacting with cholinergic receptor nicotinic alpha 7 subunit (CHRNA7) and subsequently activating the JAK2/STAT3 signaling pathway. We found that aberrant CHRNA7 expression can serve as an independent prognostic factor for ESCC patients. In multiple ESCC mouse models, dextromethorphan and metformin synergistically repressed nicotine-enhanced cancer-initiating cells (CIC) properties and inhibited ESCC progression. Mechanistically, dextromethorphan non-competitively inhibited nicotine binding to CHRNA7 while metformin downregulated CHRNA7 expression by antagonizing nicotine-induced promoter DNA hypomethylation of *CHRNA7*. Since dextromethorphan and metformin are two safe FDA-approved drugs with minimal undesirable side-effects, the combination of these drugs has a high potential as either a preventive and/or a therapeutic strategy against nicotine-promoted ESCC and perhaps other nicotine-sensitive cancer types as well.

## Introduction

Tobacco smoking is one of the most impactful risk factors for many malignancies with nicotine being the major ingredient responsible for the development of addiction to both tobacco consumption and nicotine-based e-cigarettes [1–3]. Apart from potentially causing immediate harm to adolescent health, nicotine consumption also sows the seeds for future problems by predisposing consumers to substance abuse [4, 5]. More recent evidence suggests that nicotine as well as its metabolites such as nitrosamine ketone can induce DNA damage and hinder DNA damage repair [6]. However, the carcinogenic effects of nicotine, as well as the underlying molecular mechanisms, are not fully delineated [7, 8], and this is part of the reason for a lack of effective therapeutic agents to blockade the detrimental effect of nicotine in the context of malignancy.

Nicotine exerts its biological effects mainly via the nicotinic acetylcholine receptors (nAChRs) [9, 10]. The functional nAChRs comprise homopentamers of α-subunits or heteropentamers with at least one α- and one β-subunit. In mammals, seven α subunits (*CHRNA2* to *CHRNA7*, *CHRNA9*, and *CHRNA10*), four β (*CHRNB1* to *CHRNB4*), and one each of subunits δ (*CHRND*), ε (*CHRNE*), and γ (*CHRNG*) have been identified. Multiple lines of evidence indicate that homopentameric nAChRs of CHRNA1, CHRNA3, CHRNA4, CHRNA5, CHRNA6, CHRNA7, and CHRNA9 are involved in cancer initiation and progression [11–15]. Among them, CHRNA7 and CHRNA9 have been reported to be capable of inducing cancer stem cell-like cells (CSC) or cancer-initiating cells (CIC) [16–18]. Therefore, targeting these nAChRs could potentially inhibit nicotine-induced tumor initiation.

Esophageal squamous cell carcinoma (ESCC) is the 6th leading cause of cancer-related mortality worldwide and the CICs are critical for malignancy, drug resistance, and recurrence [19–22]. Both environmental and lifestyle-related factors, such as nicotine consumption play important roles in ESCC initiation and progression [19–22]. By using ESCC as a model system with both in vitro and in vivo approaches, we demonstrated that nicotine is capable of enhancing ESCC CIC properties by activating the CHRNA7/JAK2/STAT3/SOX2 axis. More importantly, two FDA-approved drugs dextromethorphan and metformin at their pharmacological doses can synergistically inhibit nicotine-enhanced CIC properties and ESCC progression.

## Results

### Nicotine enhances tumor-initiating properties through CHRNA7

**Fig. 1:**
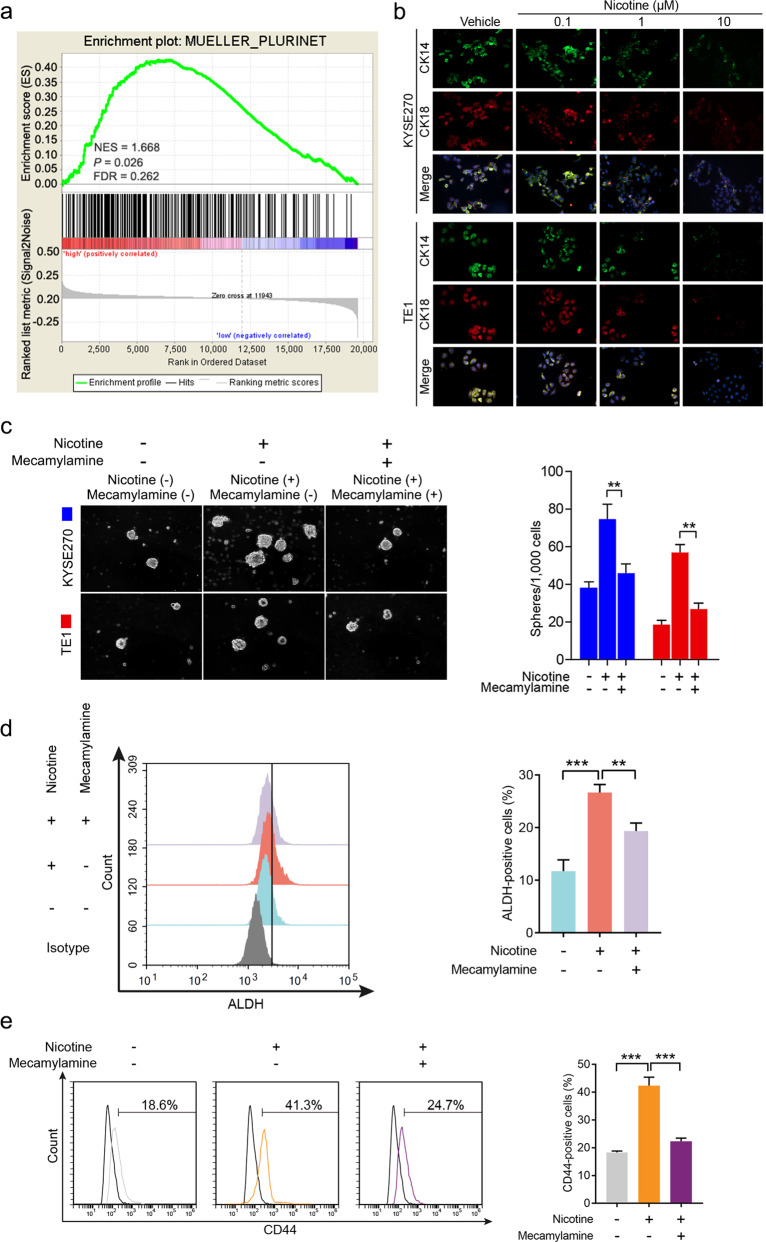
Nicotine induces ESCC CIC properties by activating nicotinic acetylcholine receptor. **a** Gene set enrichment analysis (GSEA) of enrichment of MUELLER_PLURINET in nonsmoking patients vs. smoking patients in GSE53625 dataset. FDR false-discovery rate *q* value. NES normalized enrichment score. **b** Representative images of immunofluorescence for CK14 and CK18 in KYSE270 cells (upper panel) and TE1 cells (bottom panel) treated with different concentrations (0.1, 1, and 10 μM) of nicotine for 48 h. **c** Representative images of spheres formed by KYSE270 and TE1 cells treated with nicotine alone or combined with mecamylamine (left panel). Quantification of spheres formed per 1000 cells (right panel). **d** Flow cytometry analysis of the ALDH-positive population in TE1 cells treated with nicotine or mecamylamine. **e** Flow cytometry analysis of the CD44-positive population in TE1 cells treated with nicotine or mecamylamine. Data are shown as the means of three independent experiments or representative data. Error bars indicate SD. **P < 0.01, ***P < 0.001 by Student’s *t*-test or one-way ANOVA with post hoc intergroup comparisons.

To determine if nicotine played any role in tumors other than lung cancer, we first conducted gene set enrichment analysis (GSEA) and revealed that in human ESCC the CIC signature was highly correlated with cigarette smoking (Fig. 1a). We then treated two human ESCC cell lines KYSE270 and TE1 with different concentrations (0.1, 1, and 10 μM) of nicotine, and found that treatment with nicotine decreased the levels of both cytokeratin 14 (CK14) and CK18 in a dose-dependent manner, drastically at 10 μM (Fig. 1b). In addition, we found that nicotine is also capable of increasing both the number and the size of ESCC tumorspheres in 3D culture (Fig. 1c). Since mecamylamine, an antagonist of the nAChRs, is capable of counteracting nicotine-enhanced sphere formation (Fig. 1c), we postulated that nicotine acted on ESCC through nAChRs. Flow cytometry analyses showed that nicotine treatment significantly increased percentages of two CIC markers, ALDH^+^ cells (Fig. 1d) and CD44^+^ cells (Fig. 1e), and these effects were blocked by mecamylamine (Fig. 1d, e). These observations collectively indicate that nicotine can enhance tumor-initiating properties in human ESCC cells likely through one or more nAChR.

**Fig. 2:**
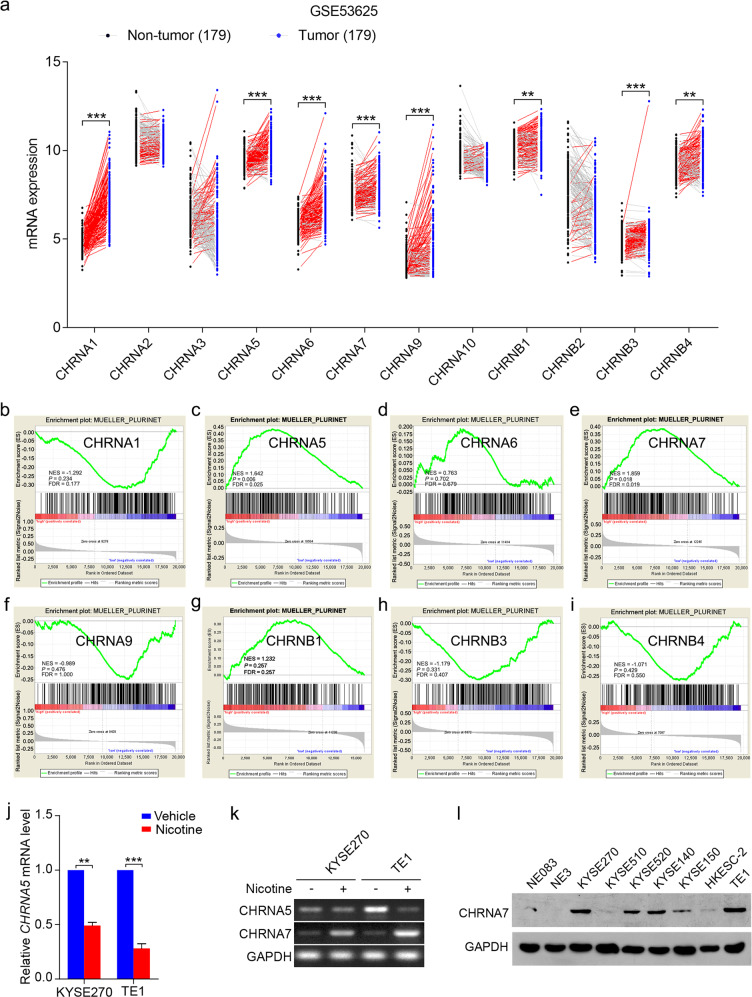
CHRNA7 is closely correlated with ESCC CIC properties. **a** The mRNA levels of the 12 nicotinic acetylcholine receptors derived from an ESCC dataset in GEO (GSE53625), which includes 179 ESCC (tumor) and their paired adjacent normal tissues (nontumor). The red and gray lines connect the nontumor and tumor values in patients with high and low expression, respectively. GSEA plots of enrichment of MUELLER_PLURINET in high expression versus low expression of CHRNA1 (**b**), CHRNA5 (**c**), CHRNA6 (**d**), CHRNA7 (**e**), CHRNA9 (**f**), CHRNB1 (**g**), CHRNB3 (**h**), CHRNB4 (**i**) in the GSE53625 dataset. FDR false-discovery rate *q* value. NES normalized enrichment score. **j** The mRNA levels of *CHRNA5* in KYSE270 and TE1 cells treated with nicotine or vehicle. **k** Representative images of semi-quantitative PCR analysis of CHRNA5 and CHRNA7 in KYSE270 and TE1 cells treated with or without nicotine. GAPDH was used as an internal loading control. **l** Immunoblotting analyses of CHRNA7 expression in ESCC cell lines and immortalized normal esophageal epithelial cell lines. Data are shown as the means of three independent experiments or representative data. Error bars indicate SD. *P < 0.05, **P < 0.01, ***P < 0.001 by paired *t*-test.

We then want to determine which subunit (s) of nAChRs is responsible for nicotine-enhanced CIC properties. Although the levels of CHRNA1, CHRNA5, CHRNA6, CHRNA7, CHRNA9, CHRNB1, CHRNB3, and CHRNB4 in ESCC were all significantly higher than that of the normal esophageal tissues (Fig. 2a), results from GSEA indicate that only CHRNA5 and CHRNA7 were associated with CIC properties in ESCC patients (Fig. 2b–i). Nevertheless, when ESCC cells were treated with nicotine, the mRNA levels of CHRNA7, but not CHRNA5, is upregulated (Fig. 2j, k). In addition, compared with that in immortalized esophageal cell lines, the protein levels of CHRNA7 are significantly higher in all the ESCC cell lines tested (Fig. 2l). Based on these observations, we postulated that CHRNA7 may play a more important role in nicotine-induced CIC properties.

**Fig. 3:**
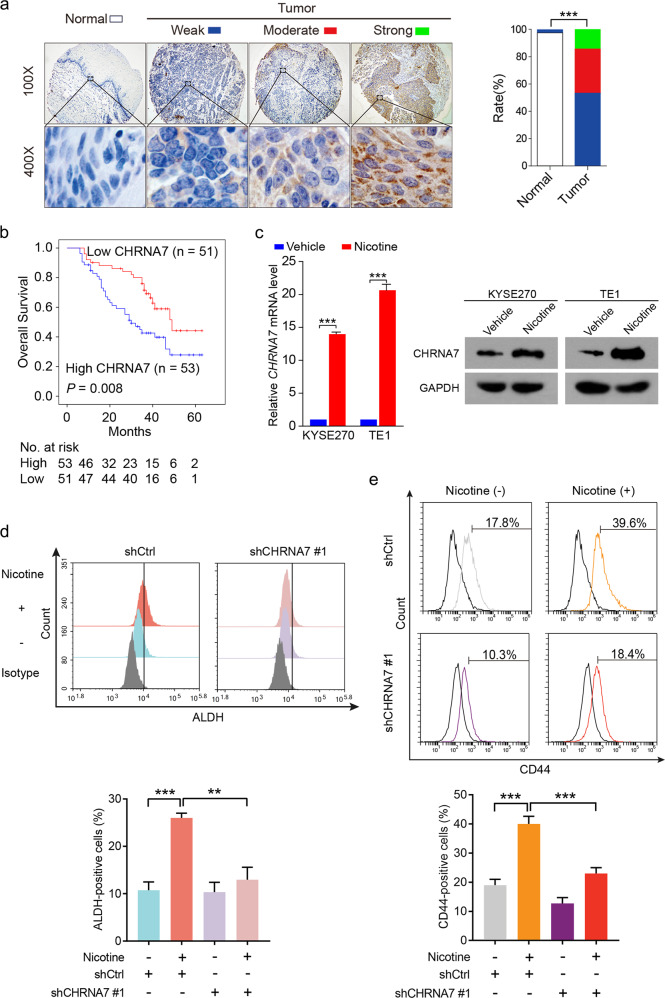
Upregulated CHRNA7 is associated with poor prognosis and correlates with CIC properties. **a** Immunohistochemistry (IHC) of CHRNA7 in 104 human ESCC tissues and their paired adjacent normal tissues (left panel). Bar charts showing the percentage of cases with different levels of CHRNA7 in tumors and their paired adjacent normal tissues (right panel). **b** Kaplan–Meier curves compared the overall survival in ESCC patients with high and low protein levels of CHRNA7. **c** RT-qPCR (left panel) and immunoblot (right panel) analysis of CHRNA7 in KYSE270 and TE1 cells treated with nicotine or vehicle. GAPDH was used as an internal control. **d** Flow cytometry analysis of ALDH-positive population in TE1 cells transfected with shCHRNA7 #1 or shCtrl were treated with or without nicotine (top panel). Histograms showing the proportion of ALDH-positive cells (bottom panel). **e** Flow cytometry analysis of CD44-positive population in TE1 cells transfected with shCHRNA7 #1 or shCtrl were treated with or without nicotine (top panel). Histograms showing the proportion of CD44-positive cells (bottom panel). Data are shown as the means of three independent experiments or representative data. Error bars indicate SD. **P < 0.01, ***P < 0.001 by Wilcoxon matched-pair signed-rank test or one-way ANOVA with post hoc intergroup comparisons.

To determine the clinical relevance of CHRNA7 in ESCC, we analyzed a tissue microarray with ESCC specimens and their non-cancerous surrounding tissues from 104 patients and found that cells in ESCC tissues expressed significantly higher levels of CHRNA7 than that of non-tumorous tissues (*P* < 0.001, Wilcoxon matched-pair signed-rank test; Fig. 3a). Receiver operator characteristic analysis identified an optimal cutoff score of 7.583 for IHC score, which categorized 50.96% (53 of 104) patients with CHRNA7 overexpression. The clinicopathological characteristics of this cohort were compared between cases with high (above cutoff score) and low (at or below cutoff score) CHRNA7 expression (Supplementary Table 1). We found that high expression of CHRNA7 is highly correlated with lymph node metastasis (*P* = 0.010), tumor stage (*P* = 0.019), and smoking history (*P* = 0.048). Notably, the CHRNA7 levels in ESCC specimens in a majority of smokers are higher than their respective non-cancerous surrounding tissues (Fisher exact test; *P* = 0.018, Supplementary Fig. 1a). Kaplan–Meier analysis revealed that patients with high CHRNA7 expression were associated with a significantly shorter overall survival (median: 34.9 months) than those with low CHRNA7 expression (median: 46.4 months) (log-rank test; *P* = 0.008, Fig. 3b). Univariate and multivariate Cox regression analyses indicate that high CHRNA7 expression is a significant prognostic factor for overall survival (hazard ratio 1.903; confidence interval 1.061–3.414; *P* = 0.031, Supplementary Table 2). Consistently, the RNAseq data derived from 95 ESCC patients in TCGA dataset (TCGA-ESCA) also showed a significant association between CHRNA7 expression and ESCC prognosis (log-rank test; *P* = 0.025, Supplementary Fig. 1b). More importantly, nicotine treatment upregulated both mRNA and protein levels of CHRNA7 in the ESCC cells (Fig. 3c). Since the expression of CHRNA7 is higher in tumorspheres (Supplementary Fig. 2a), and knocking down CHRNA7 (Supplementary Fig. 2b) not only reduced the size of the spheres but also the number of CD44^+^ cells (Supplementary Fig. 2c), we conducted flow cytometry assays and demonstrated that knocking down CHRNA7 attenuated the nicotine-induced subpopulation of both ALDH^+^ (Fig. 3d) and CD44^+^ cells (Fig. 3e). These data altogether demonstrate that CHRNA7 plays an important role in the nicotine-enhanced CIC properties of ESCC.

### Nicotine enhances CIC properties through the CHRNA7/JAK2/STAT3/SOX2 axis

The exact molecular mechanisms in CHRNA7-mediated downstream signaling pathways are far from fully defined [23, 24]. Since our GSEA analysis found that CHRNA7 expression is associated with JAK2-STAT3 signaling in different cancers (Supplementary Fig. 2d), we test the hypothesis that nicotine enhances tumor-initiating capacity by activating CHRNA7 and subsequently the JAK2/STAT3 pathway. Since knockdown and overexpression CHRNA7 in two ESCC cells reduced or enhanced phosphorylation of JAK2 (p-JAK2) and STAT3 (p-STAT3), respectively, without affecting the total levels of these factors (Supplementary Fig. 2e), we examined the correlation between CHRNA7 and CIC-associated markers in a GEO database (GSE47404) and found that CHRNA7 expression is highly associated with SOX2 (*r* = 0.6812, *P* < 0.001) and NANOG (*r* = 0.2689, *P* = 0.023) (Supplementary Fig. 2f). Given the well-established role of SOX2 in tumor-initiating cells, we experimentally demonstrated the parallel relationship between CHRNA7 and SOX2 when CHRNA7 is either knocked down or overexpressed (Supplementary Fig. 2e). Interestingly, protein–protein interaction network analysis (STRING) suggests that CHRNA7 interact with CSC associated proteins through STAT3 (Supplementary Fig. 2g). In addition, compared with control (left), strong Duolink signals in cells treated with nicotine (right) were observed in a proximity ligation assay using antibodies against CHRNA7 and JAK2 (Supplementary Fig. 2h). These data altogether suggest that nicotine-activated CHRNA7 may interact with JAK2 at first and subsequently activates the STAT3/SOX2 pathway.

**Fig. 4:**
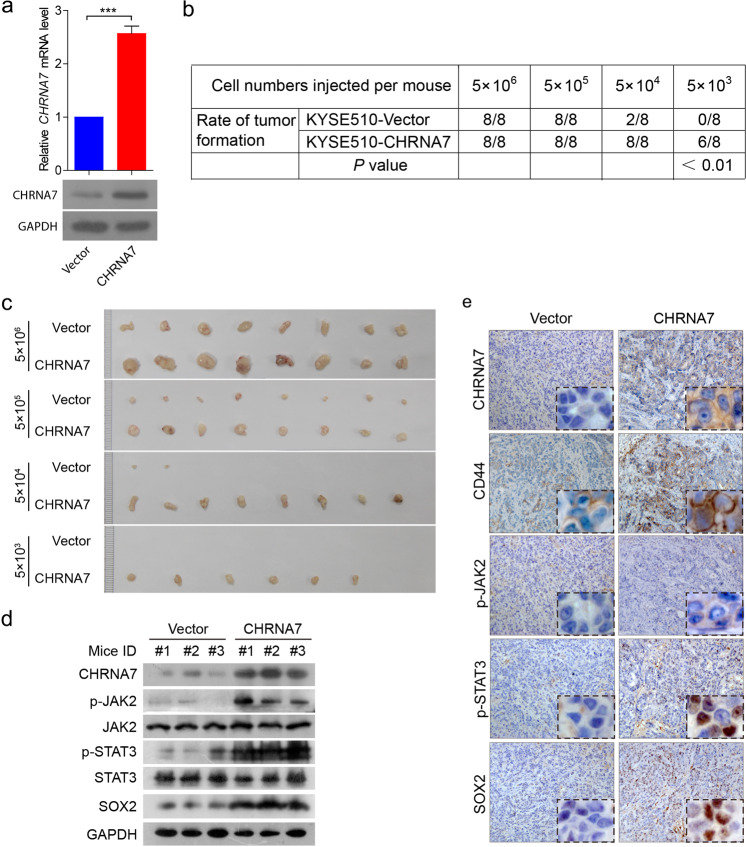
The effect of CHRNA7 on ESCC CIC properties in vivo. **a** RT-qPCR (top panel) and immunoblot (bottom panel) analysis of CHRNA7 in KYSE510 cells transfected with CHRNA7 overexpression plasmid or empty vector. GAPDH was used as an internal control. **b** The table showing that the *P* value of each case in which the nude mice (*n* = 8 per group) were injected with KYSE510 cells transfected with CHRNA7 overexpression plasmid or empty vector. **c** Tumors derived from nude mice injected KYSE510 cells stably transfected with CHRNA7-expressing plasmid or empty vector at the end of the experiments. **d** Immunoblot of CHRNA7-JAK2-STAT3-SOX2 in representative tumors derived from nude mice injected KYSE510 cells stably transfected with CHRNA7-expressing plasmid or empty vector. **e** IHC of CHRNA7-JAK2-STAT3-SOX2 in representative tumors derived from KYSE510 stably transfected with CHRNA7-expressing plasmid or empty vector. GAPDH was used as an internal control. Data are shown as the means of three independent experiments or representative data. Error bars indicate SD. ***P < 0.01 by Student’s *t*-test.

To further substantiate the role of the CHRNA7-regulated JAK2/STAT3/SOX2 axis in CIC properties, we inoculated different numbers of cells harboring overexpressed CHRNA7 (Fig. 4a) into the flank of nude mice. The overexpressed CHRNA7 was capable of not only enhancing tumor formation (Fig. 4b) but also generating bigger tumors (Fig. 4c). Notably, only CHRNA7-overexpressing cells formed tumors when a small number (5 × 10^3^) of cells were implanted (Fig. 4c). Also, western blotting (Fig. 4d) and IHC (Fig. 4e) showed that overexpressed CHRNA7 not only activated the JAK2/STAT3 pathway but also increased CIC properties evidenced by increased CD44 and SOX2. Consistent with the results from the patients (Supplementary Table 1), CHRNA7 overexpression led to an increase of metastatic lesions in the lymph node (Supplementary Fig. 3a). Interestingly, CHRNA7 overexpression enhanced the CIC properties of metastatic cells in the lymph node, as evidenced by CD44 staining (Supplementary Fig. 3b). These data altogether suggest that nicotine enhances tumor-initiating capacity in primary tumors and metastatic lesions by targeting the CHRNA7/JAK2/STAT3/SOX2 axis.

### Metformin counteracts nicotine-upregulated CHRNA7 by enhancing promoter hypermethylation

To explore whether inhibiting CHRNA7 expression or repressing its activity could be an efficacious therapeutic strategy, we first conducted bioinformatics analyses of the publicly available dataset with 95 human ESCC samples in cBio Cancer Genomics Portal (http://cbioportal.org) but failed to identify any mutation, deletion, fusion or two cases of amplification of *CHRNA7* in this dataset (Supplementary Fig. 4a). However, by analyzing the methylation status of CHRNA7 gene in two independent ESCC microarray datasets (GSE20123 and GSE52826), we found that DNA methylation of *CHRNA7* promoter in ESCC cells was significantly (*P* < 0.001 and *P* < 0.01, respectively) lower than that in the normal tissues (Supplementary Fig. 4b). Since it has been reported that metformin is capable of increasing DNA hypermethylation [25–27] and we have previously demonstrated that metformin possesses antineoplastic property toward ESCC [21, 28, 29], we decided to explore (1) if metformin can inhibit nicotine-induced CIC traits, (2) whether nicotine upregulates CHRNA7 in ESCC cells by altering promoter DNA methylation of *CHRNA7* gene, and if so, (3) whether metformin can downregulate CHRNA7 by counteracting nicotine-mediated hypomethylation.

**Fig. 5:**
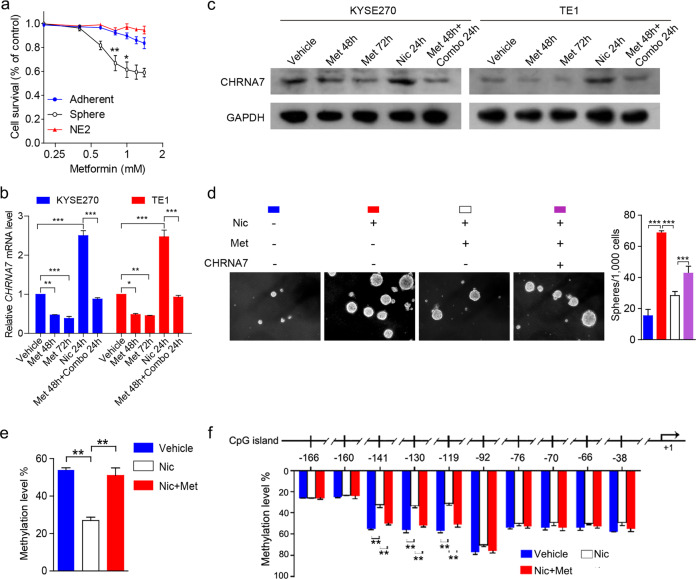
Metformin downregulates CHRNA7 by enhancing its promoter DNA hypermethylation to repress ESCC CIC properties. **a** Cell survival curves of NE2, adherent- or sphere-cultured TE1 cells treated with metformin at the indicated concentrations. **b**, **c** The mRNA and protein levels of CHRNA7 in KYSE270 and TE1 cells treated with vehicle, metformin (Met) for 48 h, Met for 72 h, nicotine (Nic) for 24 h, Met for 48 h followed by the combination of Met and Nic for 24 h. GAPDH was used as an internal control. **d** Representative images of spheres formed by TE1 cells transfected with CHRNA7 or vector treated with or without metformin. **e** The methylation level of CHRNA7 in TE1 cells treated with vehicle, Nic, Nic, and Met. **f** The methylation status on ten CpG islands in TE1 cells treated with vehicle, Nic, Nic, and Met. Data are shown as the means of three independent experiments or representative data. Error bars indicate SD. *P < 0.05, **P < 0.01, ***P < 0.001 by Student’s *t*-test or one-way ANOVA with post hoc intergroup comparisons.

We first examined the effect of metformin on nicotine-induced CIC properties of ESCC cells and immortalized non-malignant esophageal cell line (NE2) cultured in 2D and 3D conditions in the presence of different concentrations of metformin. Low concentration (between 0.6 and 1.0 mM) of metformin could inhibit the proliferation of 3D cultured cancer cells with minimal effect on either non-cancer cells or 2D cultured cancer cells (Fig. 5a). To demonstrate the role of CHRNA7 in metformin-inhibited CIC properties, we estimated the mRNA (Fig. 5b) and protein (Fig. 5c) levels of CHRNA7 in two ESCC cancer cell lines, when they were treated with metformin (0.8 mM) and nicotine alone or in combination. We found that metformin can downregulate both basal level and nicotine-enhanced CHRNA7 expression. Metformin was also capable of inhibiting CHRNA7-mediated spheroid formation and growth (Fig. 5d). These data demonstrated that pharmacologically achievable concentrations of metformin can suppress nicotine-induced CIC traits, likely through repression of CHRNA7. Next, we investigated (1) if nicotine-upregulated CHRNA7 is promoter hypomethylation-dependent, and (2) if metformin can downregulate CHRNA7 by counteracting nicotine-mediated hypomethylation. MassARRAY [26, 30] showed that nicotine downregulated DNA methylation of the *CHRNA7* promoter region significantly (*P* < 0.01), and metformin counteracted nicotine-mediated hypomethylation (Fig. 5e). Further analyses pinpointed the methylation sites were clustered between −119 and −141 above the *CHRNA7* transcription starting site (Fig. 5f). These data suggest that metformin counteracts nicotine-induced ESCC progression by blocking *CHRNA7* promoter hypomethylation.

### Dextromethorphan suppresses nicotine-induced CIC properties of ESCC by non-competitive inhibition of CHRNA7

**Fig. 6:**
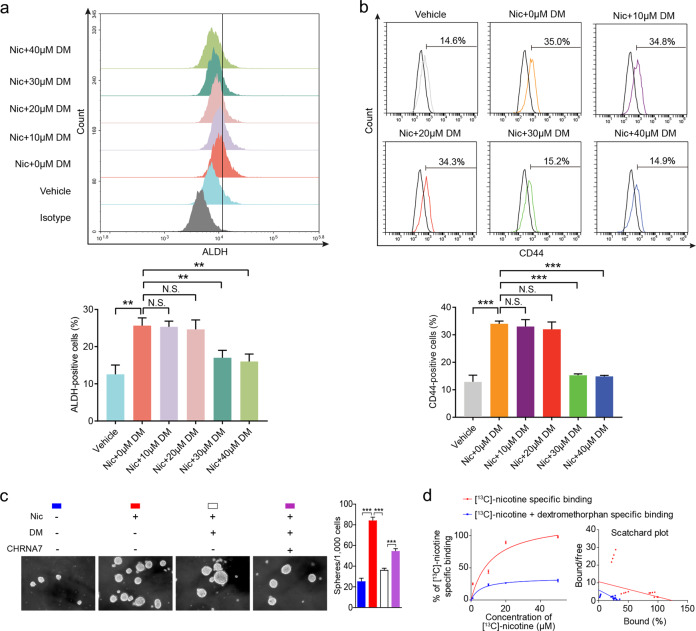
Dextromethorphan inhibits ESCC CIC properties by non-competitive inhibition of CHRNA7. **a** Flow cytometry analysis of ALDH-positive population in TE1 cells treated with vehicle, 10 μM nicotine, 10 μM nicotine and dextromethorphan (DM) (10, 20, 30, or 40 μM) for 48 h (top panel). Histograms showing the proportion of ALDH-positive cells (bottom panel). **b** Flow cytometry analysis of CD44-positive population in TE1 cells treated with vehicle, 10 μM nicotine, 10 μM nicotine, and dextromethorphan (DM) (10, 20, 30, or 40 μM) (top panel). Histograms showing the proportion of CD44-positive cells (bottom panel). **c** Representative images of spheres formed by TE1-CHRNA7 or TE1-vector treated with 10 μM nicotine and/or 30 μM dextromethorphan. **d** Saturation binding curves for [^13^C]-nicotine specific binding to HEK293 cells stably transfected with an overexpressed CHRNA7 plasmid with or without 30 µM dextromethorphan. Data are shown as the means of three independent experiments or representative data. Error bars indicate SD. **P < 0.01, ***P < 0.001 by Student’s *t*-test or one-way ANOVA with post hoc intergroup comparisons. NS non-significant.

By examining the publicly available data in DrugBank (https://www.drugbank.ca/drugs/), we found that CHRNA7 could be inhibited by dextromethorphan, one of the most widely used over-the-counter cough suppressants (Supplementary Fig. 5). It has been reported that dextromethorphan could potentially target different members of the nAChR family including CHRNA2, CHRNA3, CHRNA4, CHRNA7, and CHRNB4. But as a non-competitive antagonist of CHRNA7, dextromethorphan can attenuate nicotine’s antinociceptive and reinforcing effects at a relatively low dosage [31, 32]. We then examined if dextromethorphan can inhibit nicotine-induced ESCC tumor-initiating capacity. Two ESCC cell lines were treated with either 10 μM nicotine alone or in combination with different concentrations (10, 20, 30, or 40 μM) of dextromethorphan and found that dextromethorphan at a concentration of 30 or 40 μM is capable of counteracting nicotine-enhanced CIC properties (Fig. 6a, b). In addition, dextromethorphan (30 μM) can counteract nicotine-induced ESCC sphere formation (Fig. 6c). To understand the underlying mechanism, binding assays of [^13^C]-nicotine in overexpressed CHRNA7 HEK293 cells with or without dextromethorphan (30 µM) indicated that dextromethorphan antagonized nicotine binding as a non-competitive inhibitor (Fig. 6d).

### Metformin and dextromethorphan inhibit nicotine-induced tumor-initiating capability synergistically

**Fig. 7:**
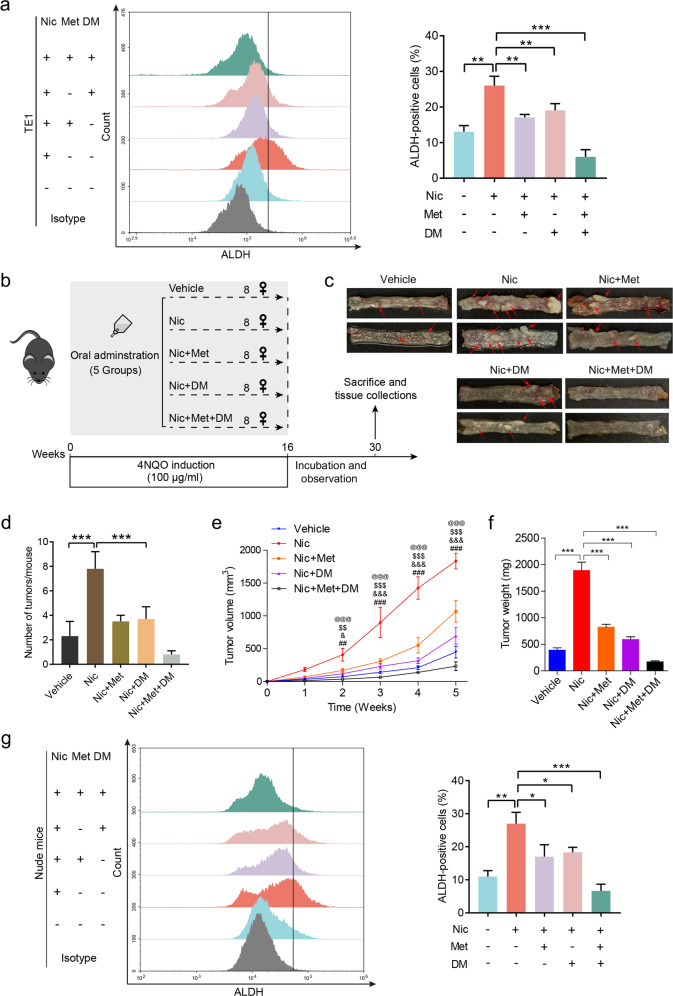
Dextromethorphan and metformin inhibit nicotine-induced ESCC CIC properties synergistically. **a** The ALDH-positive population in TE1 cells treated with vehicle, Nic, Nic and Met, Nic and DM, or Nic, Met, and DM. **b** The protocol of chemical-induction of esophageal squamous cell carcinoma in C57BL/6 mice. **c** Tumors per mouse in the esophagus from mice treated with vehicle, Nic, Nic and Met, Nic and DM, or Nic, Met, and DM. Red arrows indicate esophageal tumors. **d** The number of tumors per mouse in the esophagus from mice was plotted. Growth curves (**e**) and the weight (**f**) of tumors derived from TE1 cells in nude mice treated with vehicle, Nic, Nic and Met, Nic and DM, or Nic, Met, and DM. **g** The ALDH-positive population in tumors derived from nude mice treated with vehicle, Nic, Nic and Met, Nic and DM, or Nic, Met, and DM (left panel). Histograms showing the proportion of ALDH-positive cells (right panel). Data are shown as the means of three independent experiments or representative data. Error bars indicate SD. *P < 0.05, **P < 0.01, ***P < 0.001 by Student’s *t*-test or one-way ANOVA with post hoc intergroup comparisons. ^#^Nic vs. vehicle, ^&^Nic vs. Nic and Met, ^$^Nic vs. Nic and DM, and ^@^Nic vs^.^ Nic, Met, and DM; *n* = 8 per group; error bars indicate SD; ^##^*P* < 0.01, ^###^*P* < 0.001; ^&^*P* < 0.05, ^&&&^*P* < 0.001; ^$$^*P* < 0.01, ^$$$^*P* < 0.001; ^@@@^*P* < 0.001 by two-way ANOVA followed by a Tukey-Kramer post hoc test.

Given the different mechanisms in metformin- and dextromethorphan-counteracted nicotine-induced ESCC tumor-initiating capacity, we expected that combination of these drugs could inhibit nicotine-induced ESCC development more efficiently. Indeed, results from both sphere formation of TE1 and KYSE270 cells (Supplementary Fig. 6a) and flow cytometry (Fig. 7a, Supplementary Fig. 6b) indicate that a combination of metformin and dextromethorphan inhibited nicotine-induced CIC properties more efficiently than either of them individually. Notably, a combination of these drugs appeared to synergistically counteract nicotine-induced expression of CHRNA7, as well as downstream signaling in terms of p-JAK2, p-STAT3, and SOX2 (Supplementary Fig. 6c). To demonstrate that metformin and dextromethorphan counteract nicotine-induced CIC properties synergistically in vivo, we used two models, the 4-nitroquinoline N-oxide (4NQO)-induced ESCC mouse model and the nude mice carrying a human ESCC xenograft. Compared to single-agent treatment, co-administration of dextromethorphan, and metformin dramatically decreased the tumor formation (i.e., tumor number per mouse and individual tumor size) in nicotine-treated 4NQO-induced ESCC mice (Fig. 7b–d). IHC analysis showed that, among the experimental groups, the combination of metformin and dextromethorphan resulted in the greatest inhibition of the CHRNA7-JAK2-STAT3 axis and the maximum reduction of the CIC markers (Supplementary Fig. 6d). Either single or combination treatment, these two FDA-approved drugs exhibited minimal undesirable side-effects (Supplementary Fig. 6e). In the nude mouse model, nicotine markedly accelerated ESCC tumor growth (Fig. 7e). Although either dextromethorphan or metformin alone is capable of inhibiting nicotine-induced tumor growth to a certain degree, a combination of them almost completely ablated nicotine-enhanced cancer cell growth (Fig. 7e, Supplementary Fig. 7a) and tumor weight (Fig. 7f). Of note, IHC (Supplementary Fig. 7b) and western blotting (Supplementary Fig. 7c) showed that the combination of dextromethorphan and metformin suppressed the CHRNA7-JAK2-STAT3 axis more than either one alone in ESCC xenografts. Consistently, a combination of these drugs suppressed ALDH- (Fig. 7g) and CD44-positive cells (Supplementary Fig. 7d) synergistically. These data altogether demonstrate that a combination of dextromethorphan and metformin counteract nicotine-induced tumor initiation capability synergistically and repress nicotine-induced cancer progression efficiently.

## Discussion

**Fig. 8:**
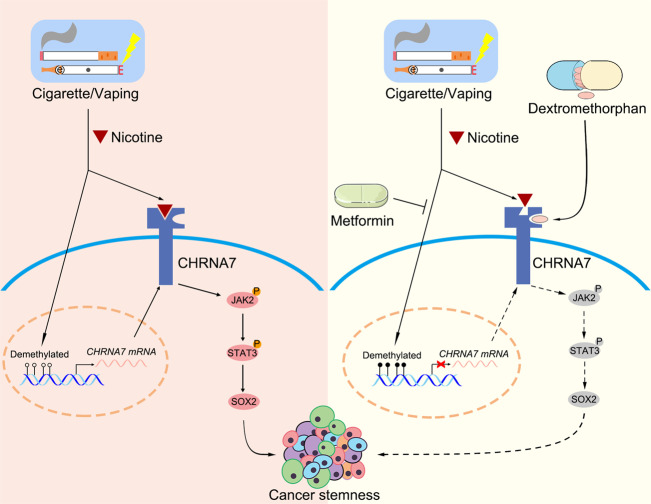
Dextromethorphan and metformin inhibit CIC properties in ESCC. Nicotine enhances CIC properties by activating the CHRNA7/JAK2/STAT3/SOX2 signaling pathway, while dextromethorphan together with metformin counteract nicotine-induced CIC properties by repressing CHRNA7/JAK2/STAT3/SOX2 signaling pathway. Mechanistically, dextromethorphan non-competitively inhibits nicotine-CHRNA7 interaction while metformin downregulates CHRNA7 by enhancing its promoter hypomethylation, and thereby they synergistically inactivating the JAK2/STAT3/SOX2 signaling pathway.

We identified a potentially effective strategy for treating nicotine-induced cancer growth and progression using two FDA-approved drugs, dextromethorphan and metformin, with long safety records. We used ESCC, nicotine-related cancer, as a model system to demonstrate that the combinatory therapy synergistically inhibits nicotine-enhanced CIC properties by targeting CHRNA7. Our studies reveal that CHRNA7, a nicotine-enhanced receptor, confers high oncogenic potential by inducing CIC properties and serves as a predictor of poor prognostic in ESCC. We have provided multiple lines of evidence derived from two ESCC mouse models, patient sample analysis, bioinformatics analyses, and cellular studies to indicate that CHRNA7 is the specific receptor subtype responsible for nicotine-enhanced CIC properties in ESCC. Our results derived from bioinformatics analyses, in vivo and in vitro studies and clinical data indicate that CHRNA7 mediates nicotine-induced ESCC tumor-initiating capacity at least in part via activating the JAK2/STAT3/SOX2 signaling pathway, which has been proven to be frequently dysregulated in human ESCC [33]. Accordingly, using dextromethorphan and metformin together is an effective strategy to block CHRNA7/JAK2/STAT3/SOX2 signaling (Fig. 8).

Given that *CHRNA7* lacks genetic alterations, such as amplification but is rich in DNA methylation in its promoter, we conducted a series of in vitro and in vivo experiments proving that metformin can inhibit nicotine-induced CIC traits by counteracting nicotine-mediated DNA hypomethylation of the *CHRNA7* promoter. Indeed, our results are supported by other reports that metformin is capable of enhancing DNA hypermethylation in the breast, endometrial, and ovarian cancer cells [26, 27, 34]. In contrast to metformin, dextromethorphan, one of the most widely used over-the-counter cough suppressants [35], has not been used in any anti-cancer studies. However, dextromethorphan has been found to benefit patients with Alzheimer’s disease and mitigate pain in painful diabetic neuropathy and chemotherapy-induced peripheral neuropathy [36–38]. Indeed, results from our animal model demonstrated that metformin and dextromethorphan alone can counteract nicotine-mediated ESCC progression effectively. Interestingly, metformin and dextromethorphan acted synergistically and almost completely abrogated nicotine-mediated CIC properties and progression. To our knowledge, we are the first to experimentally demonstrate that (1) metformin and dextromethorphan can downregulate the expression and inhibit the activity of CHRNA7, respectively, and (2) combination of these reagents can counteract nicotine-enhanced ESCC tumor-initiating capacity, and therefore inhibit ESCC progression.

In summary, our findings support the prognostic and therapeutic importance of CHRNA7 in nicotine-induced cancer-initiating properties, by showing that a combination of dextromethorphan and metformin may serve as either a preventive (e.g., secondary prevention) or therapeutic strategy against nicotine-relevant cancers.

## Materials and methods

### Cells and reagents

The human ESCC cell lines HKESC-2 and three human immortalized esophageal epithelial cells (NE2, NE3 and NE083) were kindly provided by Dr. SW Tsao (University of Hong Kong, China). The human ESCC cells TE1 cells were kindly provided by Dr. XC Xu (M.D. Anderson Cancer Center, Houston, TX, USA). The human ESCC cells KYSE140, KYSE150, KYSE510, KYSE270, and KYSE520 cells were obtained from the tumor cell bank of the Chinese Academy of Medical Science. These ESCC cells were cultured in DMEM or RPMI 1640 medium supplemented with 10% FBS 37 °C in a humidified atmosphere with 5% CO_2_. Immortalized NE2, NE3 and NE083 cells were cultured in Defined Keratinocyte-SFM medium (DK-SFM, Gibco). All cell lines have been authenticated using short tandem repeat DNA profiling (Beijing Microread Genetics Co., Ltd., China). Nicotine and metformin were purchased from Sigma-Aldrich. Dextromethorphan was purchased from MedChemExpress. [^13^C]-nicotine was purchased from Cambridge Isotope Laboratories, Inc.

### Gene set enrichment analyses

The ESCC dataset GSE53625 from GEO, breast invasive carcinoma dataset, lung adenocarcinoma dataset, and colon adenocarcinoma/Rectum adenocarcinoma Esophageal carcinoma dataset from TCGA database were analyzed using GSEA software (Version 2.2.1, http://software.broadinstitute.org/gsea/index.jsp).

### [^13^C]-Nicotine binding assay

Cells were cultured to 90% confluence and scraped. The cells were then incubated in [^13^C]-nicotine alone or with dextromethorphan at room temperature for 30 min followed by 60 min at 4 °C. After incubation, cells were washed three times using ice-cold PBS, vacuum dried and then wrapped with a silver-cup. Samples were measured on Thermo Scientific MAT253 (HT-IRMs). For the saturation binding assay, 1, 10, 20, and 50 µM concentrations of [^13^C]-nicotine, with or without 30 µM of dextromethorphan, were used to test the dissociation constant K_d_ and the maximal binding capacity B_max_.

### 4NQO-induced carcinogenesis model

Six-week-old C57BL/6 mice (Vital River Lab Animal Technology Co Ltd., Beijing, China) were given 100 μg/mL carcinogen 4NQO (Cat. N8141, Sigma) in drinking water for 16 weeks to induce ESCC. The mice were randomized into five groups (*n* = 8 per group). To investigate the effects of nicotine, metformin and dextromethorphan on ESCC, nicotine (200 μmol/L), metformin (160 mg/kg), or dextromethorphan (40 mg/kg) was taken orally for 16 weeks and then the mice were euthanized 12 weeks later. Animals were randomly assigned to groups and no blinding was done. All the animal experiments were approved by the Ethics Committee and the Chancellor’s Animal Research Committee at SUMC (SUMC2014-148).

### Statistical analysis

All statistical analyses except for microarray data were carried out using the statistical software package SPSS 17.0 (SPSS, Inc., Chicago, IL, USA). The comparisons between two groups were performed with Student’s *t*-tests and those among >2 groups with one-way ANOVA with post hoc intergroup comparisons. The correlation between CHRNA7 expression and clinicopathologic data of patients was analyzed with the Pearson *χ*^2^ test. Survival curves were plotted with the Kaplan–Meier method and compared by the log-rank test. Survival data were evaluated by univariate and multivariate Cox regression analyses. The correlations of the histoscore between CHRNA7 and the indicated biomarkers of ESCC were determined by Spearman’s rank test. *P* value of less than 0.05 was considered statistically significant.

Additional details about materials and methods are described in Supplementary Data.

## Supplementary information

Supplementary data
