# Correction: Repurposing dextromethorphan and metformin for treating nicotine-induced cancer by directly targeting CHRNA7 to inhibit JAK2/STAT3/SOX2 signaling

**DOI:** 10.1038/s41388-021-01936-w

**Published:** 2021-07-23

**Authors:** Lu Wang, Liang Du, Xiao Xiong, Yusheng Lin, Jianlin Zhu, Zhimeng Yao, Shuhong Wang, Yi Guo, Yuping Chen, Kyla Geary, Yunlong Pan, Fuyou Zhou, Shegan Gao, Dianzheng Zhang, Sai-Ching Jim Yeung, Hao Zhang

**Affiliations:** 1 Department of General Surgery, The First Affiliated Hospital of Jinan University, and Institute of Precision Cancer Medicine and Pathology, School of Medicine, Jinan University, Guangzhou, Guangdong China; 2 Department of Biomedical Sciences of Cells & Systems, Section Molecular Cell Biology and Radiation Oncology, University Medical Center Groningen, University of Groningen, Groningen, The Netherlands; 3 Department of Hematology, University Medical Center Groningen, University of Groningen, Groningen, The Netherlands; 4 Endoscopy Center, Affiliated Cancer Hospital of Shantou University Medical College, Shantou, Guangdong China; 5 Department of Thoracic Surgery, Affiliated Cancer Hospital of Shantou University Medical College, Shantou, Guangdong China; 6 Department of Bio-Medical Sciences, Philadelphia College of Osteopathic Medicine, 4170 City Avenue, Philadelphia, PA 19131 USA; 7 The Fourth Affiliated Hospital of Henan University of Science and Technology, Anyang, 455001 Henan China; 8 Department of Thoracic Surgery, Anyang Tumor Hospital, Anyang, 455001 Henan China; 9 College of Clinical Medicine, The First Affiliated Hospital of Henan University of Science and Technology, Henan Key Laboratory of Cancer Epigenetics, Luoyang, 471003 China; 10 Department of Emergency Medicine, University of Texas MD Anderson Cancer Center, Houston, TX USA; 11 Department of Endocrine Neoplasia and Hormonal Disorders, University of Texas MD Anderson Cancer Center, Houston, TX USA

**Keywords:** Cancer prevention, Drug development, Targeted therapies, Oesophageal cancer, Oncogenes

Correction to: *Oncogene* 10.1038/s41388-021-01682-z, published online 18 February 2021

Only after the article was published online did the authors notice the misspelling of the second author’s name. It should be “Liang Du” instead of “Du Liang”. The authors sincerely apologize for any inconvenience this might have caused.

The original article has been corrected.

