# Supplementary material for: Repurposing dextromethorphan and metformin for treating nicotine-induced cancer by directly targeting CHRNA7 to inhibit JAK2/STAT3/SOX2 signaling
Source: Oncogene. 2021 Feb 18;40(11):1974–87. doi: 10.1038/s41388-021-01682-z (PMC7979537; doi:10.1038/s41388-021-01682-z)
Supplement: Supplementary file 1 — Supplementary data [file 41388_2021_1682_MOESM1_ESM.docx]

**Supplementary Data**

**Supplementary Materials and Methods**

*Clinical specimens*: A total of 104 paraffin-embedded specimens were histopathologically and clinically diagnosed as ESCC and obtained from the Affiliated Tumor Hospital of Shantou University Medical College. Patients who underwent preoperative radiotherapy or chemotherapy for ESCC were excluded from this study. Clinical research protocols of this study were reviewed and approved by the Ethics Committee of Shantou University Medical College (IRB serial number: # 04–070). Written informed consents were obtained from patients in accordance with principles expressed in the Declaration of Helsinki.

*Immunohistochemistry*: 4μm sections from the formalin-fixed paraffin-embedded clinical specimens, 4NQO-induced esophageal tumor tissues or the tumor xenograft tissues were processed and stained with antibodies against CHRNA7 (Cat. No. 8367012, Biolegend), CD44 (Cat. No. 157107, Abcam; Cat. No. 3570, Cell Signaling Technology), SOX2 (Cat. No. 23064, Cell Signaling Technology)，p-JAK2 (Y1007+Y1008) (Cat. No. 32101, Abcam), p-STATA3(Tyr705) (Cat. No. 9145, Cell Signaling Technology), or CK-pan (Cat. No. 4545, Cell Signaling Technology). Sections immunostained with rabbit IgG or mouse IgG as the primary antibody were used as negative controls, known IHC positive slide was used as a positive control. The percentage of positively stained was scored using the following scales: 0, no staining of cells in any field; 1, ≤ 10%; 2, 11-50%; 3, 51-75%; 4, > 75%. The intensity of staining was scored using the following scales: 1+, weak staining; 2+, moderate staining; 3+, strong staining. Percentage (P) and intensity (I) of nuclear or cytoplasm or membrane expression were multiplied to generate a numerical score (S = P • I).

*Cell proliferation assays*: Cells were were seeded into 96-well plates at a density of 2000 cells/100 uL and treated with metformin (0.2, 0.4, 0.6, 0.8, 1.0, 1.2, 1.4, 1.6, 1.8, 2.0 mM) for 48 hours. Then CCK-8 solution (Cat: HY-K0301, MedChem Express) was added to each well at the endpoint. After incubation for 3 hours, the optical density (OD) values were measured with a microplate reader (Bio-Tek) at 450 nm.

*Sphere formation assays*: 500 cells were seeded in 6-well ultra-low attachment plates (Corning). Cells were cultured in serum-free DMEM/F12 medium (Gibco) supplemented with 1xN2 (Life Technologies), 10 ng/ml epidermal growth factor (EGF, PeproTech), 10 ng/ml basic fibroblast growth factor (bFGF, PeproTech) and 5 μg/mL insulin (Sigma Aldrich). After 10~14 days, spheres were monitored under a microscope.

*Flow cytometry analysis*: Cells were dissociated with trypsin, resuspended at a concentration of 1 × 10^4^/mL in PBS, and stained with anti-CD44 antibody (Cat. No. 15-0441, eBioscience) and ALDH kit (Cat. No. 01700, Stem Cell Technologies). The cells were detected by BD AccuriTMC6 Flow Cytometer (BD Biosciences). The data were analyzed by Flowjow software and obtained from 3 independent experiments.

*Cell transfection*: The full-length coding sequences of CHRNA7 were cloned into the pEZ-Lv201 plasmid. The shRNA targeting CHRNA7 was cloned into psi-LVRU6GP plasmid. The plasmid carrying CHRNA7 and the plasmid carrying shCHRNA7 were obtained from GeneCopoeia. To induce overexpression of CHRNA7 in KYSE510 or HEK 293 cells, the cells were stably transfected with the plasmid carrying CHRNA7. For inhibition of CHRNA7 expression in TE1 cells, the cells were stably transfected with the plasmid shCHRNA7. All transfections were performed according to the manufacturer’s instructions using Lipofectamine 3000 (Life Technologies).

*Immunofluorescence analysis*: Cells were grown on glass coverslips, fixed in 4% PFA for 20 min. The cells were stained with rabbit anti-CK14 antibody (Cat. No. ab53115, Abcam), and mouse anti-CK18 antibody (Cat. No. ab668, Abcam) overnight at 4°C. Secondary antibodies were incubated with donkey anti-rabbit Alexa-Fluor-594 (Red), and Alexa-Fluor-488 (Green)-conjugated donkey anti-mouse antibodies. Cells were mounted in ProLong™ Gold Antifade Mountant with DAPI (P36935, Invitrogen). Samples were examined under a confocal fluorescence microscope (Olympus, Japan).

*The quantitative real-time PCR assay*: Total RNA was extracted from the cells using TRIzol (Thermo Fisher). 2 μg RNA was reverse transcribed using High Capacity cDNA Reverse Transcription Kit (Applied Biosystems, USA) according to the manufacturer instructions. Then an equal amount of cDNA was amplified and quantified by using SYBR Green PCR amplification kit (Thermo Fisher) in the Applied Biosystems 7500 system as described previously . The cDNA was subjected to semi-quantitative PCR and quantitative real-time PCR (RT-qPCR) with the following primers: *CHRNA7* forward: 5’-AACCTGCTGTACATCGGCTT-3’ and reverse: 5’-GAAGACCGAGAAGGCCATGAG-3’; *CHRNA5* forward: 5’-GACTTATGATGGATCACAGGTTGA-3’ and reverse: 5’-ATAAAAGAGAGGCAGGCGCT-3’; *GAPDH* forward: 5’-TGCACCACCAACTGCTTAGC-3’ and reverse: 5’-GGCATGGACTGTGGTCATGAG-3’.

*Western blot analysis*: Proteins in the lysates of the cultured cells or tissues were separated on SDS-PAGE, transferred onto the PVDF membranes. The membranes were incubated with primary antibodies against CHRNA7 (Cat. No. 8367012, Biolegend), JAK2 (Cat. No. 3230, Cell Signaling Technology), p-JAK2 (Y1007+Y1008) (Cat. No. 32101, Abcam), STAT3 (Cat. No. 12640, Cell Signaling Technology), p-STAT3 (Tyr705) (Cat. No. 9145, Cell Signaling Technology), SOX2 (Cat. No. 23064, Cell Signaling Technology) and GAPDH (Cat. No. 5174, Cell Signaling Technology) followed by HRP-conjugated secondary antibodies. Protein bands were visualized with SuperSignal West Pico Luminol/Enhancer Solution (Thermo Scientific).

*Proximity ligation assay*: The Proximity Ligation Assay (PLA) kit (Duolink®using PLA®Technology, Sigma–Aldrich) was employed to detect the interaction between CHRNA7 and JAK2 . In brief, cells grown on glass coverslips were fixed with 4% paraformaldehyde followed by blocking in 5% BSA. The cells were then incubated with mouse anti-CHRNA7 and rabbit anti-JAK2 antibodies and followed by incubation with secondary plus and minus probes, PLA–anti-(rabbit IgG), and PLA–anti-(mouse IgG). The ligation solution was added followed by an amplification solution. The PLA signals were visualized under ZEISS LSM800 confocal fluorescence microscope (ZEISS, Germany).

*DNA methylation*: Genomic DNA (500ng ~1g) was obtained from each sample and was bisulfite-converted using the EZ DNA Methylation Kit (Zymo Research, Irvine, CA, USA). DNA from cultured cells were evaluated for methylation level. Quantitative methylation analysis was performed using the Sequenom MassARRAY EpiTYPER technology platform with MassCLEAVE chemistry (Sequenom, San Diego, CA, USA), according to the manufacturer’s protocol. A 204-base pairs (bp) amplicon was amplified from the CHRNA7 gene by polymerase chain reaction with gene-specific primers: CHRNA7-forward, 5'- TTTGTTTTGTTTGGTTGGTAAGATT -3' and CHRNA7 -reverse, 5'- AAAACCAAACTCTACCTCTCCCTTA -3' using bisulfite-treated genomic DNAs as templates. The enriched amplicon, comprised of four cytosine–phosphate–guanine (CpG) sites, is located in the promoter region of the CHRNA7 gene. An initial denaturation step of 95°C for 4 min was followed by amplification for 45 cycles of 95°C for 20 s, 60°C for 30 s and 72°C for 1 min and a final elongation step of 72°C for 3 min. PCR products were treated with shrimp alkaline phosphatase. After in-vitro transcription and RNase cleavage using a Sequenom MassCLEAVE T-cleavage kit, the samples were desalted using a cation exchange resin (Sequenom) and spotted onto SpectroCHIP II matrix chips using a MassARRAY nano dispenser. Mass determination was performed with the MassARRAY Analyzer Compact MALDI-TOF mass spectrometer. Methylation calls were performed using the EpiTYPER software version 4.0, which generates quantitative results for each CpG site or an aggregate of multiple CpG sites. A mean methylation value of three CpG sites (CpG1, CpG2 and CpG4) was calculated from each sample, and the results of CHRNA7 promoter methylation were compared with those of CHRNA7 protein expression for the corresponding samples. Methylation rates >10% were defined as high methylation.

*Tumorigenicity and serial transplantation assay in nude mice*: Nude mice 4 to 5 weeks old were purchased from Beijing Vital River Laboratory Animal Technology. KYSE510-vector or KY510-CHRNA7 cells (5 ×10^3^, 5 ×10^4^, 5 ×10^5^, 5 ×10^6^, respectively) in 100 μL medium mixed with 100 μL Matrixgel™ Basement Membrane Matrix (BD Biosciences) was injected subcutaneously into the flank of each mouse (eight mice per group). The mice were observed for tumor growth every day over 6 to 8 weeks and then sacrificed by cervical dislocation. Animals were randomly assigned to groups and no blinding was done. All the animal experiments were approved by the Ethics Committee and the Chancellor’s Animal Research Committee at SUMC (SUMC2014-148).

*Tumor Xenografts*: 2 × 10^6^ TE1 cells were resuspended in 100 μL PBS and injected into the flanks of 4-week-old nude mice. The mice were randomized into five groups (n = 8 per group). All groups were treated orally with nicotine (200 μmol/L), metformin (160 mg/kg) or dextromethorphan (40 mg/kg). Tumor volumes were measured every week, and tumor mass was calculated by the following formula: volume = 0.5236 × length × width ^2^. Animals were randomly assigned to groups and no blinding was done. All the animal experiments were approved by the Ethics Committee and the Chancellor’s Animal Research Committee at SUMC (SUMC2014-148).

**Supplementary Figures and legends**

**
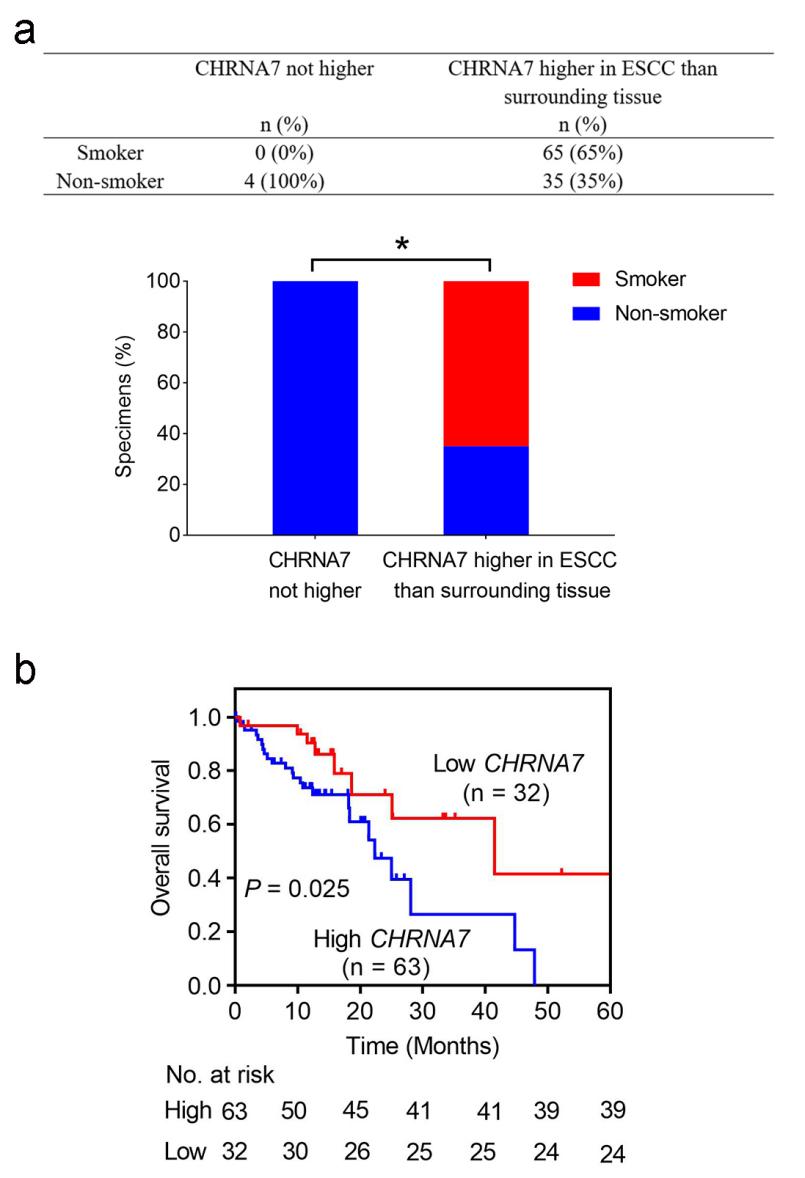
**

**Supplementary Figure 1. CHRNA7 is associated with cancer progression.** (**a**) CHRNA7 levels positively correlated with smoking status in ESCC tumors from 104 patients. (**b**) Kaplan-Meier survival analysis of TCGA dataset (TCGA-ESCA) from 95 ESCC specimens according to high and low CHRNA7 expression levels. **P* < 0.05 by Fisher exact test.

**
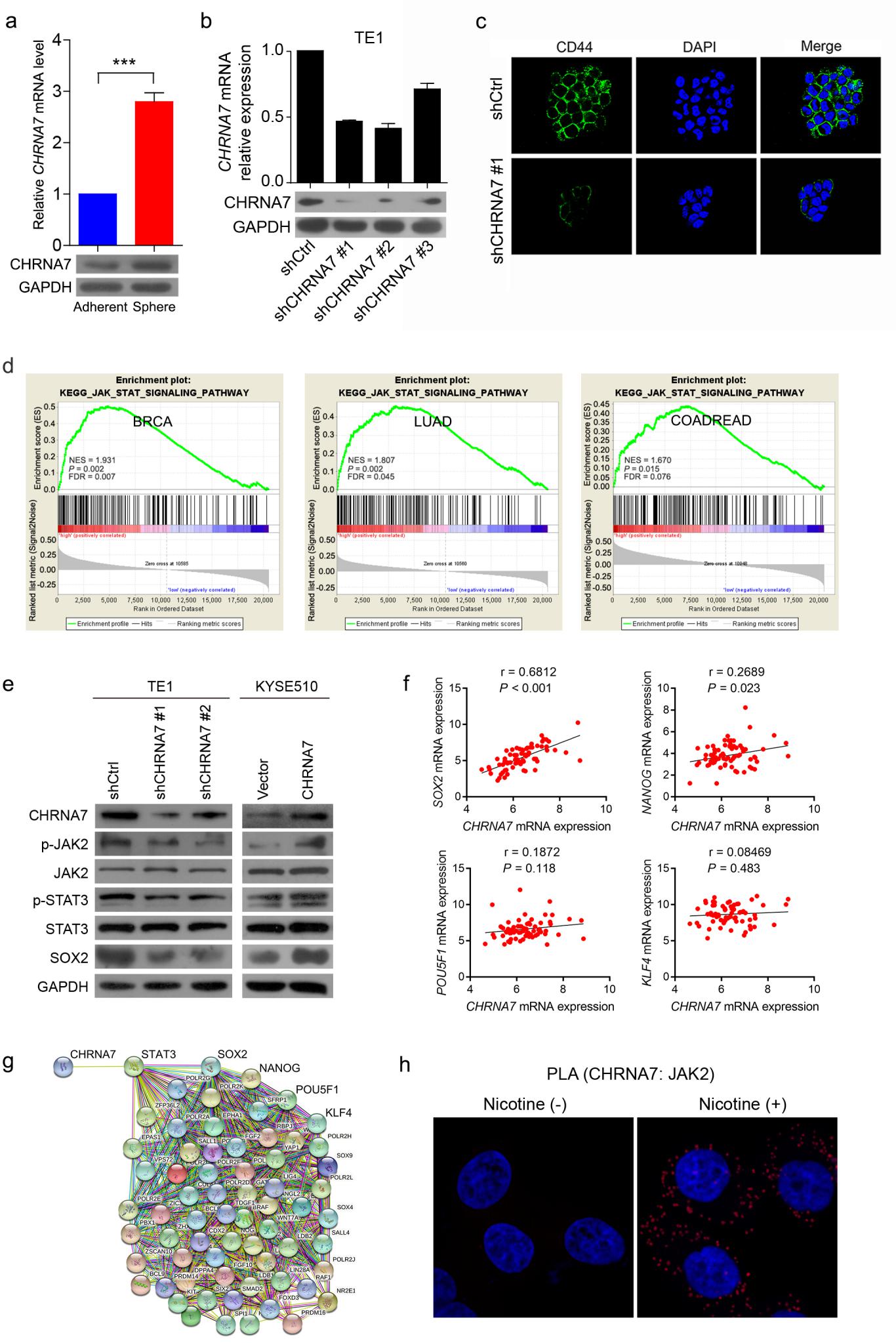
**

**Supplementary Figure 2.** **Nicotine enhances ESCC CIC properties through activating the of CHRNA7-JAK2-STAT3 axis.** (**a**) RT-qPCR (top panel) and immunoblot (bottom panel) of CHRNA7 expression in the adherent- and sphere-cultured TE1 cells. GAPDH was used as an internal control. (**b**) RT-qPCR (top panel) and immunoblot (bottom panel) of CHRNA7 expression in TE1 cells transfected with either control shRNA (shCtrl) or shRNA against CHRNA7 (#1, #2 or #3). GAPDH was used as an internal control. (**c**) Immunofluorescent staining of CD44 in TE1 cells transfected with shCHRNA7 #1 or shCtrl. CD44 was labeled in green and nuclei were stained with DAPI (blue). (**d**) GSEA plots of the patients with BRCA, LUAD and COADREAD showing positive correlations between CHRNA7 and the JAK2-STAT3 pathway (KEGG_JAK_STAT_SIGNALING_PATHWAY). (**e**) Immunoblot of the indicated CHRNA7-JAK2-STAT3 axis in TE1 cells with CHRNA7-depleted (left panel) and KYSE510 cells with CHRNA7 overexpressed (right panel). GAPDH was used as an internal control. (**f**) Pearson’s correlations of *CHRNA7* and *SOX2*, *CHRNA7* and *NANOG*, *CHRNA7* and *POU5F1*, *CHRNA7* and *KLF4* in GEO database (GSE47404). (**g**) Protein-protein interactions network analysis (STRING <https://string-db.org/>) showing that CHRNA7 was linked to the CSC pathway (GO_SOMATIC_STEM_CELL_POPULATION_MAINTENA). (**h**) Proximity Ligation Assay (PLA) detection of CHRNA7-JAK2 interaction in TE1 cells treated with or without nicotine. Data are shown as the means of three independent experiments or representative data. Error bars indicate SD. ****P* < 0.001 by Student's t-test.


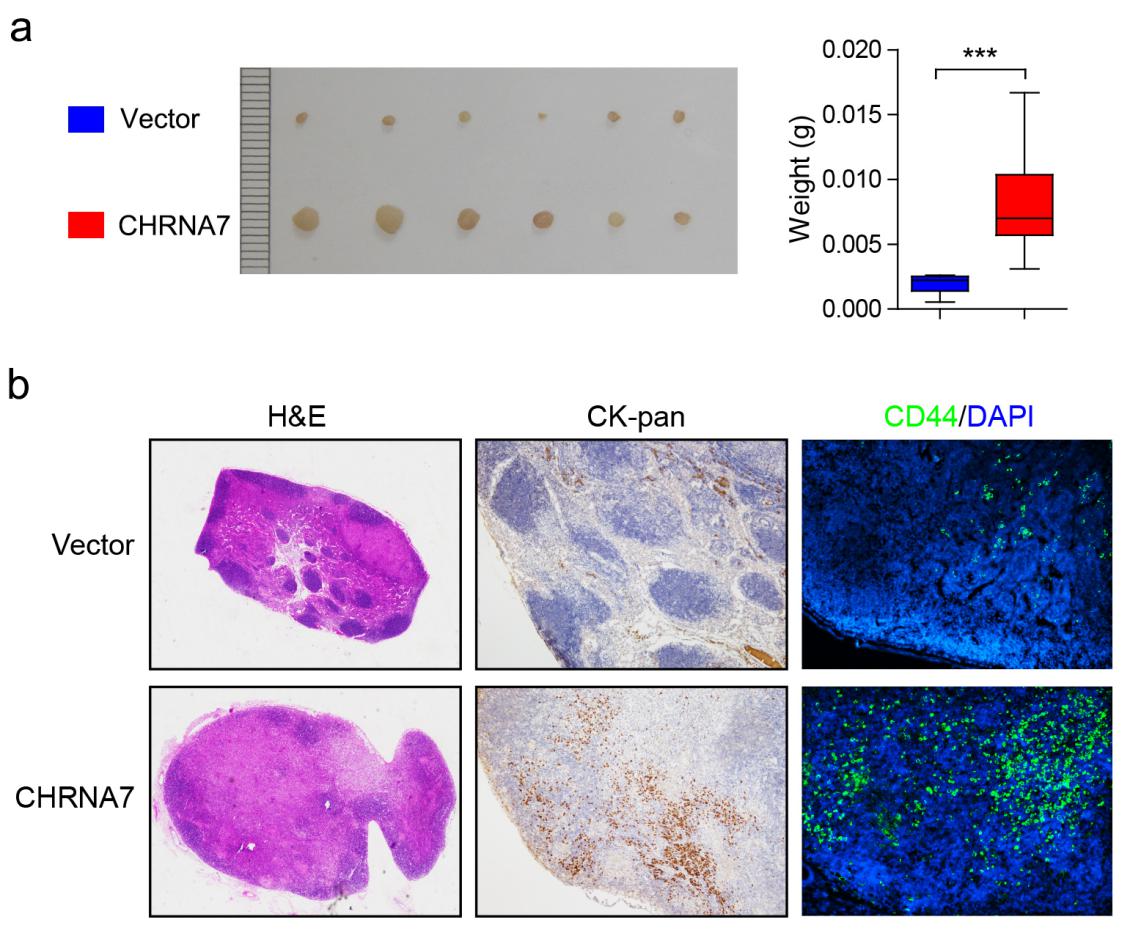


**Supplementary Figure 3. CHRNA7 enhances ESCC lymphatic metastasis.** (**a**) The inguinal lymph nodes were established by injected subcutaneously KYSE510 cells stably transfected with CHRNA7-expressing plasmid or empty vector into the flanks of nude mice. Representative images (top panel) and the weights of inguinal lymph nodes (bottom panel). (**b**) Inguinal lymph nodes were analyzed by H&E, CK-pan staining or CD44 staining. Data are shown as the means of three independent experiments or representative data. Error bars indicate SD. ****P* < 0.001 by Student's t-test.

**
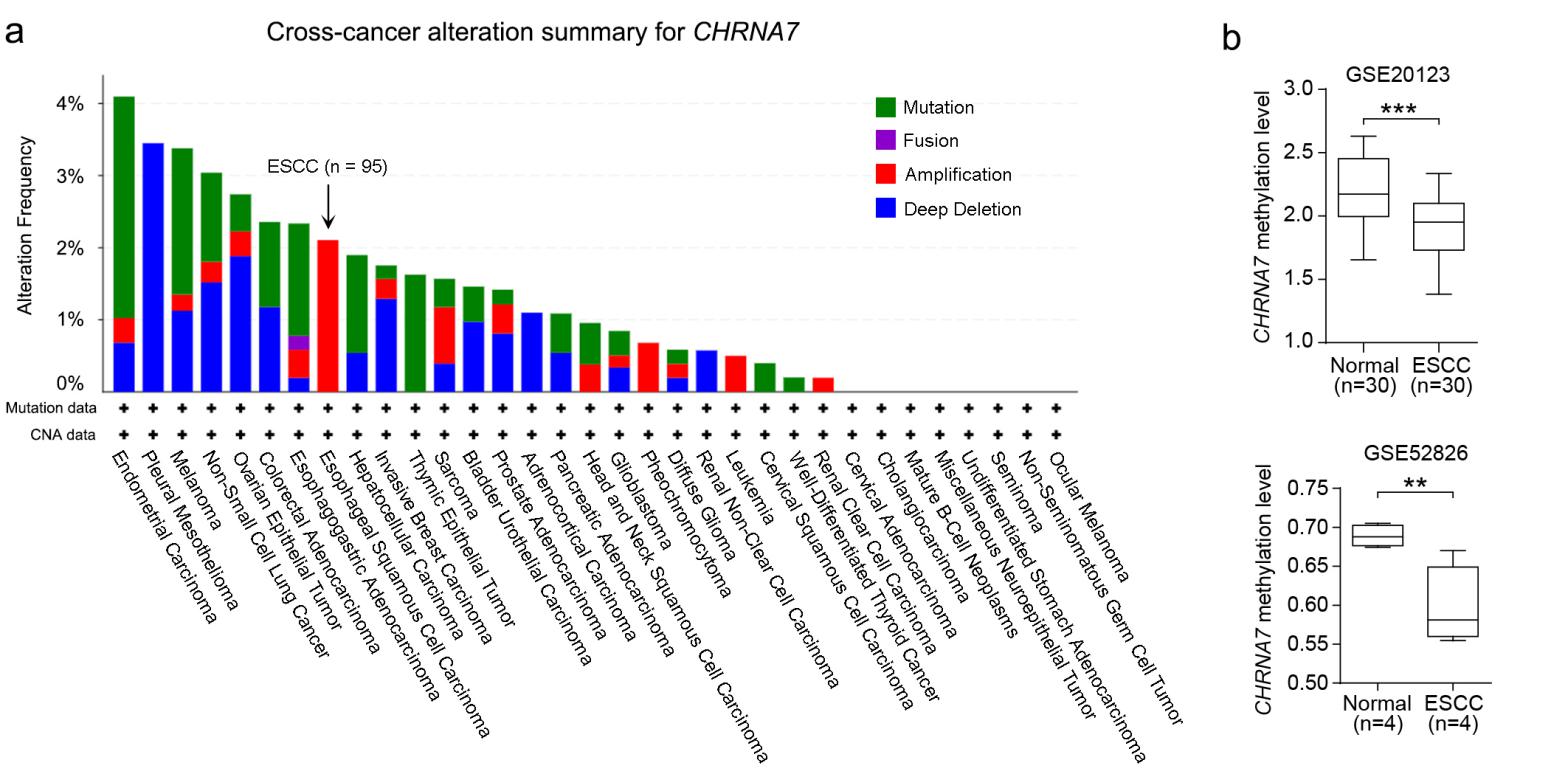
**

**Supplementary Figure 4. The promoter of CHRNA7 is hypomethylation in ESCC.** (**a**) Cross-cancer alteration of CHRNA7 with data derived from the cBioPortal for Cancer Genomics (<http://www.cbioportal.org>). (**b**) The methylation status in the promoter region of CHRNA7 gene with data derived from the ESCC dataset in GEO (GSE20123 and GSE52826). Error bars indicate SD. ***P* < 0.01, ****P* < 0.001 by Student's t-test.


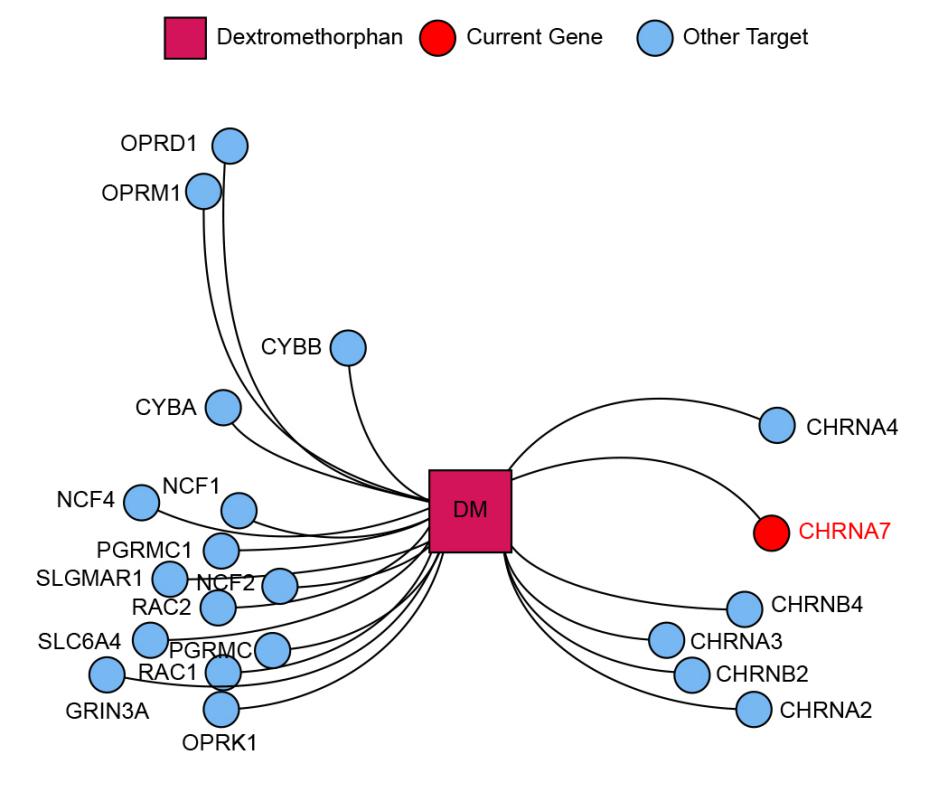


**Supplementary Figure 5. CHRNA7 is a target of dextromethorphan.** The potent targets of dextromethorphan (DM) were predicted using the DrugBank database.

**
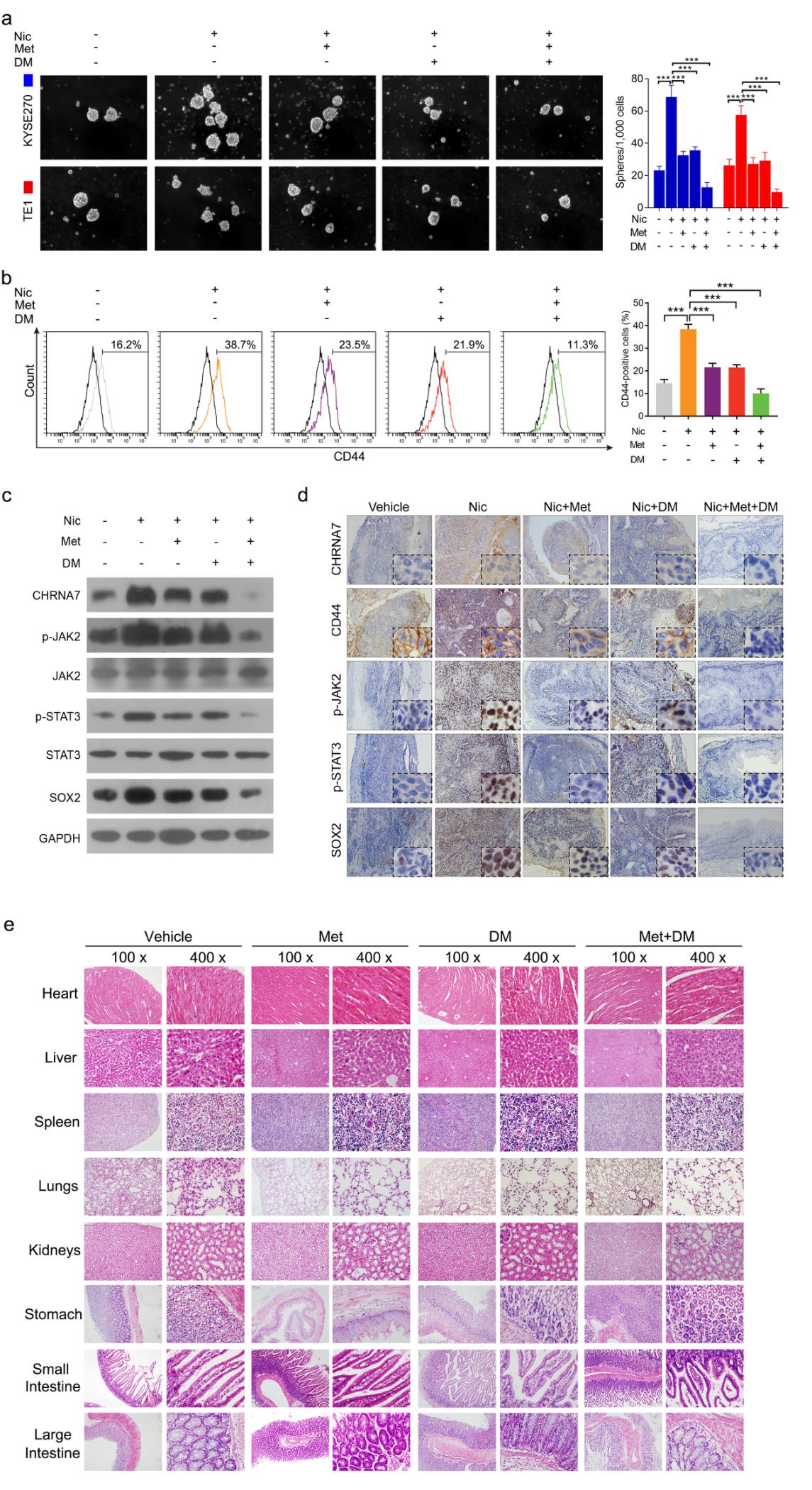
**

**Supplementary Figure 6.** **Metformin and** **dextromethorphan synergistically inhibit nicotine-induced ESCC CIC properties*.*** (**a**) Representative images of spheres formed by KYSE270 and TE1 cells treated with vehicle, Nic, Nic and Met, Nic and DM, or Nic, Met and DM. (**b**) Flow cytometry of CD44-positive population in TE1 cells treated with vehicle, Nic, Nic and Met, Nic and DM, or Nic, Met and DM. (**c**) Immunoblot of CHRNA7, p-JAK2, JAK2, p-STAT3, STAT3 and SOX2 in TE1 cells treated with vehicle, Nic, Nic and Met, Nic and DM, or Nic, Met and DM. GAPDH was used as an internal control. (**d**) IHC of CHRNA7, CD44, p-JAK2, p-STAT3 and SOX2 in esophageal tumor derived from C57BL/6 mice. (**e**) H&E of multiple organs derived from C57BL/6 mice models. Data are shown as the means of three independent experiments or representative data. Error bars indicate SD. ****P* < 0.001 by Student's t-test or or one-way ANOVA with post hoc intergroup comparisons.

**
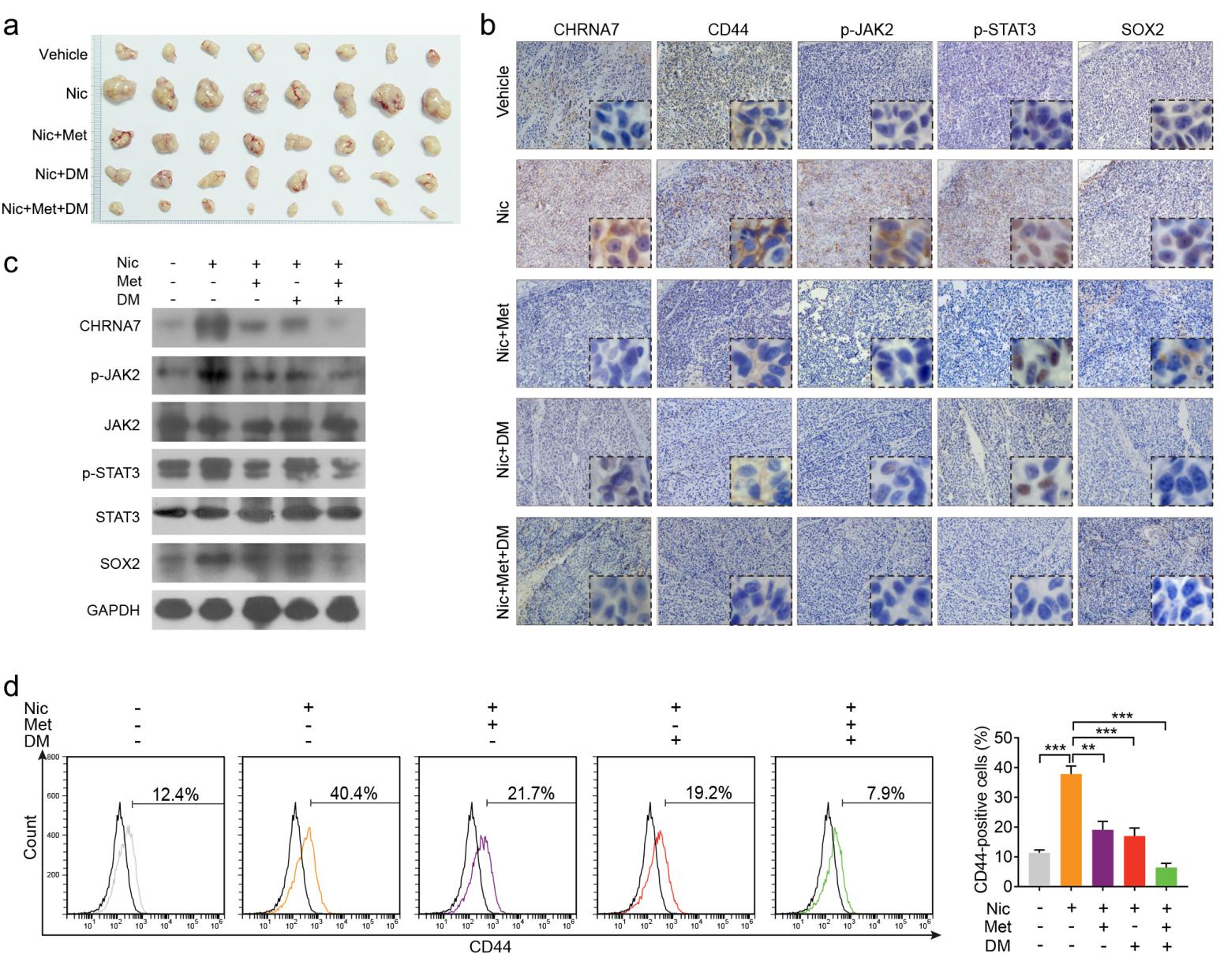
**

**Supplementary Figure 7. Metformin and dextromethorphan synergistically inhibit nicotine-induced ESCC tumor progression in animal model.** (**a**) The tumors derived from nude mice models at the end of experiments (n = 8 per group). (**b-c**) IHC and immunoblot of CHRNA7, CD44, p-JAK2, p-STAT3 and SOX2 in tumors derived from nude mice models. GAPDH was used as an internal control. (**d**) The CD44-positive population in tumors derived from nude mice models (left panel). Histograms showing the proportion of CD44-positive cells (right panel). Data are shown as the means of three independent experiments or representative data. Error bars indicate SD. ***P* < 0.01, ****P* < 0.001 by Student's t-test or or one-way ANOVA with post hoc intergroup comparisons.

**Supplementary** **Tables**

**Supplementary** **Table 1.** The relationship between the levels of CHRNA7 in ESCC tissues and the clinicopathologic characteristics.

| Variables |  | CHRNA7 expression | |  |
| --- | --- | --- | --- | --- |
|  | n | Low: n (%) | High: n (%) | *P*^a^ |
| Patients | 104 | 51 (49.0) | 53 (51.0) |  |
| Age (years) |  |  |  |  |
| ≤ 60 | 98 | 49 (50.0) | 49 (50.0) | 0.428 |
| > 60 | 6 | 2 (33.3) | 4 (66.7) |  |
| Gender |  |  |  |  |
| Male | 74 | 37 (50.0) | 37 (50.0) | 0.758 |
| Female | 30 | 14 (46.7) | 16 (53.3) |  |
| Histological differentiation |  |  |  |  |
| Well | 21 | 11 (52.4) | 10 (47.6) | 0.935 |
| Moderately | 68 | 33 (48.5) | 35 (51.5) |  |
| Poorly | 15 | 7 (46.7) | 8 (53.3) |  |
| Tumor depth |  |  |  |  |
| T_1_/T_2_ | 19 | 11 (55.8) | 8 (44.2) | 0.393 |
| T_3_/T_4_ | 85 | 40 (81.0) | 45 (19.0) |  |
| Lymph node metastasis |  |  |  |  |
| N0 | 6 | 6 (100.0) | 0 (0.0) | 0.010 |
| N1 | 98 | 45 (45.9) | 53 (54.1) |  |
| Stage |  |  |  |  |
| I/II | 45 | 28 (57.9) | 17 (42.1) | 0.019 |
| III/IV | 59 | 23 (47.1) | 36 (52.9) |  |
| Smoker |  |  |  |  |
| No | 39 | 24 (61.5) | 15 (38.5) | 0.048 |
| Yes | 65 | 27 (41.5) | 38 (58.5) |  |

^a^*P* value by chi-square test.

High CHRNA7 is defined when a tissue IHC score >7.583; the remaining individuals were considered a low CHRNA7 group.

**Supplementary** **Table 2.** Univariate and multivariate Cox proportional hazards model predicting survival in ESCC patients.

| Variables | Univariate analysis | | Multivariate analysis | |
| --- | --- | --- | --- | --- |
|  | HR (95% CI) | *P* | HR (95% CI) | *P* |
| Age (years) |  |  |  |  |
| > 60 vs. ≤ 60 | 1.776 (0.706 to 4.468) | 0.223 | 1.739 (0.684 to 4.426) | 0.245 |
| Gender |  |  |  |  |
| Male vs. Female | 1.512 (0.794 to 2.880) | 0.209 | 1.583 (0.753 to 3.327) | 0.226 |
| Histological differentiation |  |  |  |  |
| Well vs. Moderately /poorly | 1.087 (0.545 to 2.166) | 0.813 | 0.715 (0.344 to 1.486) | 0.369 |
| Stage |  |  |  |  |
| III/IV vs. I/II | 2.133 (1.193 to 3.812) | 0.011 | 2.171 (1.141 to 4.130) | 0.018 |
| Smoker |  |  |  |  |
| Yes vs. No | 1.232 (0.697 to 2.178) | 0.473 | 1.103 (0.560 to 2.175) | 0.777 |
| CHRNA7 expression |  |  |  |  |
| High vs. Low | 2.080 (1.190 to 3.638) | 0.010 | 1.903 (1.061 to 3.414) | 0.031 |

HR*,* hazard ratio; CI*,* conﬁdence interval.

High CHRNA7 is defined when a tissue IHC score >7.583; the remaining individuals were considered a low CHRNA7 group.
